# Obesity, Dietary Fats, and Gastrointestinal Cancer Risk-Potential Mechanisms Relating to Lipid Metabolism and Inflammation

**DOI:** 10.3390/metabo14010042

**Published:** 2024-01-10

**Authors:** Kathleen A. J. Mitchelson, Fiona O’Connell, Jacintha O’Sullivan, Helen M. Roche

**Affiliations:** 1 Nutrigenomics Research Group, UCD Conway Institute, UCD Institute of Food and Health, and School of Public Health, Physiotherapy and Sports Science, University College Dublin, D04 H1W8 Dublin, Ireland; 2 Department of Surgery, Trinity St. James’s Cancer Institute and Trinity Translational Medicine Institute, St. James’s Hospital and Trinity College Dublin, D08 W9RT Dublin, Ireland; 3 Institute for Global Food Security, School of Biological Sciences, Queens University Belfast, Belfast BT9 5DL, UK

**Keywords:** obesity, adipose, diet, saturated fatty acids, monounsaturated fatty acids, gastrointestinal cancer, metabolism, inflammation

## Abstract

Obesity is a major driving factor in the incidence, progression, and poor treatment response in gastrointestinal cancers. Herein, we conducted a comprehensive analysis of the impact of obesity and its resulting metabolic perturbations across four gastrointestinal cancer types, namely, oesophageal, gastric, liver, and colorectal cancer. Importantly, not all obese phenotypes are equal. Obese adipose tissue heterogeneity depends on the location, structure, cellular profile (including resident immune cell populations), and dietary fatty acid intake. We discuss whether adipose heterogeneity impacts the tumorigenic environment. Dietary fat quality, in particular saturated fatty acids, promotes a hypertrophic, pro-inflammatory adipose profile, in contrast to monounsaturated fatty acids, resulting in a hyperplastic, less inflammatory adipose phenotype. The purpose of this review is to examine the impact of obesity, including dietary fat quality, on adipose tissue biology and oncogenesis, specifically focusing on lipid metabolism and inflammatory mechanisms. This is achieved with a particular focus on gastrointestinal cancers as exemplar models of obesity-associated cancers.

## 1. Overview: Obesity, Dietary Fats, and Cancer

Global obesity rates have tripled since the 1970s. The causal relationship between obesity and several metabolic co-morbidities, including insulin resistance (IR), type 2 diabetes (T2D), and cardiovascular disease (CVD), is well characterised [1]. Indeed, obesity has emerged as a major determinant of some cancers, overtaking smoking as a leading cause [2]. There are 14 types of cancer linked to obesity, including gastrointestinal cancers such as oesophageal, gastric, liver, and colorectal cancer [3]. Importantly, obesity is a very heterogeneous condition, the impact of which, in terms of the associated metabolic and inflammatory phenotypes, differs greatly between individuals. For example, for an equivalent body weight or body mass index (BMI), some people are profoundly insulin resistant at a given body weight/adiposity, while others remain insulin sensitive [4]. Diet may be one of the driving factors contributing to these differences in metabolic phenotypes, disease, and subsequent cancer incidence, progression, and therapeutic response. Indeed, diet is an important modulator of ‘metabolic inflammation’, a cellular phenomenon wherein the metabolic configuration of an immune cell determines and drives the nature of the inflammatory response [5]. Work to date shows that saturated fatty acids (SFA) and monounsaturated fatty acids (MUFA) have differential effects on metabolism and inflammation, and thus potentially directly impact the subsequent disease risk [6]. A further exploration of the interaction between dietary fats, obesity, and metabolic inflammation within the context of gastrointestinal cancers is necessary. A better understanding is urgently required to understand how different dietary components may regulate metabolic inflammation within the context of obesity-driven cancers. This will allow for a more complete understanding of the potential role that obese adipose tissue and precision nutrition approaches have on metabolic inflammation in gastrointestinal cancer oncogenesis.

## 2. Obesity-Related Metabolic Triggers and Gastrointestinal Cancer

**Figure 1:**
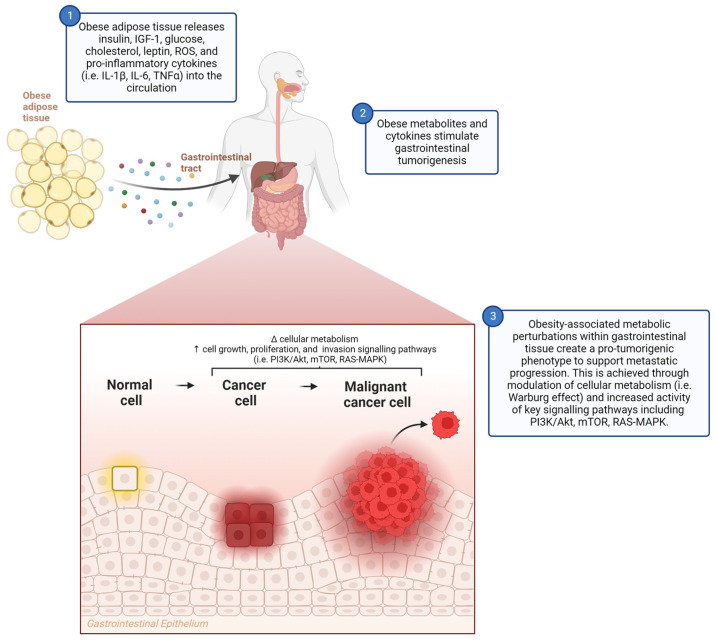
Obesity alters the immunometabolic landscape to support gastrointestinal cancer. Obese adipose tissue secretes metabolites that have been proven to show oncogenic potential. These include insulin, insulin-like growth factor (IGF-1), glucose, cholesterol, reactive oxygen species (ROS), and pro-inflammatory cytokines. These metabolites and cytokines then enter the circulation to neighbouring gastrointestinal organs where they drive normal cells to cancer cells and ultimately to metastatic cancer cells. IL, interleukin; MAPK, mitogen-activated protein kinase; mTOR, mechanistic target of rapamycin; PI3K, phosphoinositide-3-kinase; RAS, rat sarcoma; TNFα, tumour necrosis factor alpha. This figure was created using Biorender.com (accessed on 7 August 2023).

Obesity-associated metabolic dysfunction and chronic low-grade inflammation predispose people to metabolic disease development, including T2D, non-alcoholic fatty liver disease (NAFLD), and CVD [7]. Excess body fat elicits several metabolic characteristics, including hyperinsulinaemia, IR, hyperglycaemia, hypercholesterolaemia, elevated non-esterified fatty acid (NEFA, or free fatty acid) levels, and elevated triacylglycerol (TAG) levels [8], which may have oncogenic implications. These metabolic abnormalities can drive tumorigenesis through dysregulation in multiple signalling pathways (Figure 1). In obesity, plasma insulin increases with glucose levels due to the heightened insulin secretion, paired with decreased insulin clearance [9,10]. Insulin is oncogenic through activation of the phosphoinositide-3-kinase (PI3K)/Akt signalling pathway, which increases carcinogenesis in breast and colon cancer cells [11]. The PI3K/Akt signalling pathway acts by the PI3K enzyme activating Akt which subsequently activates target proteins, the main one being the serine/threonine kinase mechanistic target of rapamycin (mTOR), to promote cellular growth, proliferation, and invasion [12]. The PI3K/Akt downstream effectors concerning oncogenesis have previously been reviewed [12]. mTOR regulates cell growth through the phosphorylation of targets which control protein anabolism, growth factor signalling, and nutrient metabolism [13]. An increase in insulin receptor expression is a poor prognostic factor in lung, breast, and colon cancer [14,15,16]. Activation of the insulin receptor initiates the downstream activation of PI3K/Akt, mTOR, and rat sarcoma (RAS)-mitogen-activated protein kinase (MAPK) pathways, all of which are associated with cell survival and proliferation [17]. The RAS–MAPK pathway is common in human cancer through the aberrant activation of receptor tyrosine kinase or through gain-of-function mutations primarily seen in the *RAS* gene [18]. The activation of the MAPK cascade increases cell proliferation, differentiation, and motility through diverse mechanisms, and these were reviewed extensively [18]. Insulin-like growth factor 1 (IGF-1) signalling is also implicated in cancer development. IGF-1 increased the proliferation in oesophageal adenocarcinoma (OAC) cells and was higher in the serum of viscerally obese OAC patients [19]. Additionally, IGF-1 levels are higher in the serum of colorectal cancer (CRC) patients [20]. IR is characterised by insulin-dependent tissues being unable to take up and utilise glucose efficiently via glucose transporter type 4 (GLUT4) [21], resulting in hyperglycaemia. Warburg first observed that increased blood glucose was associated with tumorigenesis [22]. The Warburg effect occurs in proliferating cells and tumours where the glucose uptake rate increases, paired with lactate generation even when there is normal mitochondrial function and ample oxygen availability [23]. This is thought to allow for quick adenosine triphosphate (ATP) synthesis, an increase in biosynthetic pathways and cell signalling, and the disruption of tissue architecture, all enhancing tumorigenesis. The excess blood glucose seen in obesity supports the increased energetic demand of cancer cells. This is achieved through multiple mechanisms including increased insulin/IGF-1, pro-inflammatory cytokines, and pro-survival Akt/mTOR signalling [24].

Cancer cells require cholesterol for membrane synthesis and cholesterol metabolites are required for cell proliferation, migration, and invasion. Excess cholesterol increases intestinal stem cell proliferation to promote tumorigenesis [25]. Additionally, obese adipose tissue also secretes other pro-tumorigenic hormones including estrogen, leptin, reactive oxygen species (ROS), and cytokines [26]. Ultimately, obesity leads to a plethora of metabolic abnormalities with oncogenic capabilities, which could drive tumorigenesis through multiple cellular signalling pathways (Figure 1).

### 2.1. Adipose Tissue Heterogeneity and Inflammation

Heterogeneous obesity phenotypes are caused by differences in adipose location, structure, variable adipocyte, and immune cell infiltration and function [27]. There are two main adipose depots, subcutaneous adipose tissue (SAT) and visceral adipose tissue (VAT). It is proposed that VAT is the main source of obesity-driven inflammation, releases more fatty acids, and develops higher IR in comparison to SAT, providing a greater risk for developing metabolic dysfunction [28,29]. VAT’s proximity to the gastrointestinal organs, specifically the stomach, liver, oesophagus, and colon, may make it particularly problematic. Preclinical and human studies show that the depot origin dictates the lipid metabolism, with VAT displaying higher lipolysis and lipogenesis in comparison to SAT [30,31,32]. Additionally, subpopulations of adipocytes show heterogeneity through their response to external stimuli, including tumour necrosis factor alpha (TNFα), insulin, and human growth hormone [33]. Sexual dimorphism and genetic variance play a role in body fat distribution. Women store adipose tissue predominantly subcutaneously versus viscerally, while men are the opposite [34]. Transcriptomic studies show VAT and SAT adipocytes have many genetic differences in developmental genes and other resident cell populations [35,36]. However, whether gene differences are the cause or consequence of fat distribution patterns is unclear. VAT is known to be more deleterious than SAT in metabolic disease [37]; however, their roles in cancer are less evident.

#### 2.1.1. SFA and MUFA in Adipose Tissue Distribution

**Figure 2:**
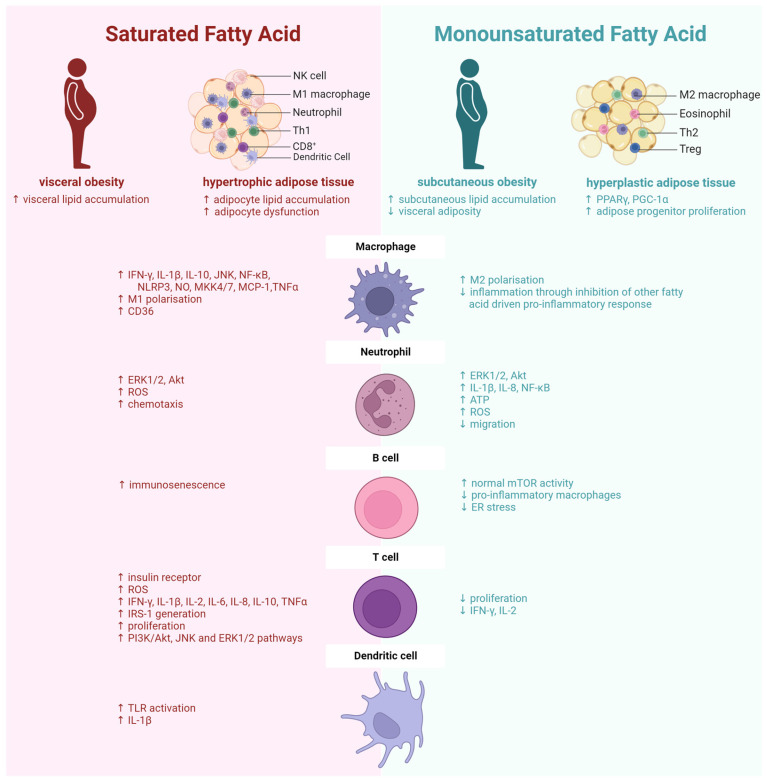
Fatty acids affect adipose distribution morphology and immune cell behaviour. Saturated and monounsaturated fatty acids can dictate adipose tissue distribution and adipocyte size (hypertrophy versus hyperplastic) [41]. Additionally, fatty acids can differentially regulate immune cell behaviour including macrophages, neutrophils, B cells, T cells, and dendritic cells. ATP, adenosine triphosphate; ER, endoplasmic reticulum; ERK, extracellular signal-regulated kinase; IFN-γ, interferon-gamma; IL, interleukin; IRS-1, insulin receptor substrate 1; JNK, c-Jun N-terminal kinase; MCP-1, monocyte chemoattractant protein-1, MKK, mitogen-activated protein kinase kinase; mTOR, mechanistic target of rapamycin; NF-κB, nuclear factor kappa B; NLRP3, NOD-, LRR- and pyrin domain-containing protein 3; NK, natural killer; NO, nitric oxide; PGC-1α, peroxisome proliferator-activated receptor gamma coactivator 1-alpha; PI3K, phosphoinositide-3-kinase; PPARγ, peroxisome proliferator-activated receptor gamma; ROS, reactive oxygen species; Th, helper T cell; TLR, toll-life receptor; TNFα, tumour necrosis factor alpha; Treg, regulatory T cell; ↑, increase; ↓, decrease. This figure was created using Biorender.com (accessed on 7 August 2023).

Diet may play an important role in adipose distribution (Figure 2). Within the human diet, the predominant SFA is palmitate, while the major MUFA is oleate. Interestingly, pre-adipocytes from human VAT and SAT displayed differential lipid accumulation following treatment with palmitate versus oleate. Acute feeding studies showed that feeding palmitate increases the lipid accumulation in VAT to a greater extent than in SAT in young men, while oleate increases lipid accumulation in SAT and not in VAT [38]. In humans, the replacement of a SFA-rich diet with MUFA showed a decrease in body and fat mass without a decrease in total energy or fat intake [39]. Furthermore, a MUFA-rich diet reduced the visceral adiposity compared to other fatty acids [40]. Ultimately, SFA-rich diets drive a more visceral adiposity, which is linked to more metabolic disease and possible tumorigenic opportunities.

#### 2.1.2. Adipose Tissue Morphology

**Figure 3:**
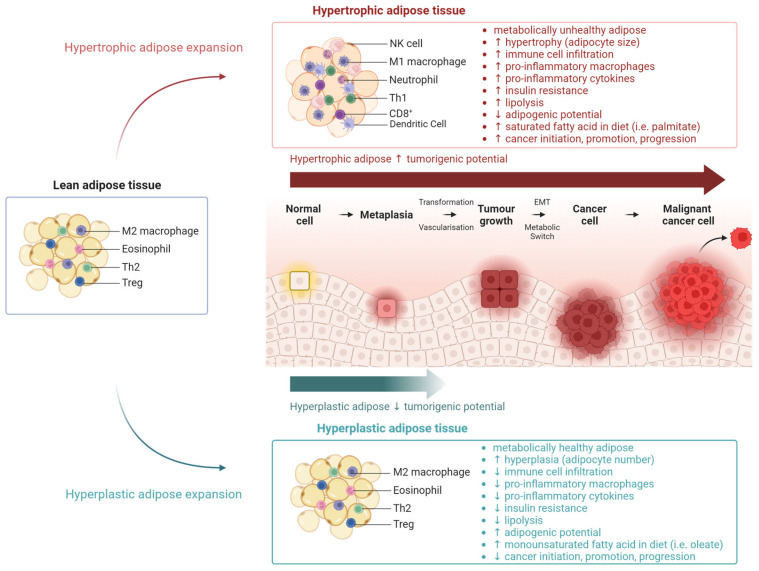
Adipose structure and function drive tumorigenesis. Overconsumption results in lean adipose tissue expansion to either hypertrophic metabolically unhealthy adipose tissue or hyperplastic metabolically healthy adipose tissue. The metabolically unhealthy adipose creates a pro-tumorigenic environment with increased immune cell infiltration, pro-inflammatory cytokine secretion, and increased free fatty acid release, which drive progression from a benign epithelium toward tumour growth and ultimately metastasis to a greater extent than metabolically healthy adipose. EMT, epithelial–mesenchymal transition; Th, helper T cells; NK, natural killer; Treg, regulatory T cells; ↑, increase; ↓, decrease. This figure was created using Biorender.com (accessed on 7 August 2023).

Adipose morphology or adipocyte architecture also affects the functionality of adipose tissue. Thus, the mechanism through which adipocytes and adipose tissue expand can also dictate metabolic health, and therefore also cancer (Figure 3). Adipogenesis is the process through which adipocytes develop from stem cells and accumulate in adipose tissue. Adipose tissue can expand through an increase in the existing adipocyte size (hypertrophy), or new adipocyte formation (hyperplasia). Hypertrophic adipose tissue is more metabolically unhealthy with an increase in insulin resistance and inflammation independent of BMI [42,43,44]. Alternatively, hyperplastic adipose tissue can be characterised as metabolically healthy, containing smaller adipocytes and reduced blood vessels. The adipogenic potential is disrupted within obese Individuals; this is ascribed in part to the inflammatory cytokine milieu, resulting in lower adipogenic gene expression leading to the formation of larger adipocytes, which are associated with IR, inflammation, and redox stress [45,46,47].

#### 2.1.3. The Impact of SFA and MUFA on Adipose Morphology

Fatty acid composition also has differential effects on adipose expansion. Pre-clinical studies show that feeding a SFA high-fat diet results in a hypertrophic adipose profile, compared to feeding a MUFA high-fat diet, despite an equal weight gain and adipose tissue weight [41] (Figure 2). This observation was mediated via the differential interleukin (IL)-1β-induced expression of peroxisome proliferator-activated receptor gamma (PPARγ) and peroxisome proliferator-activated receptor gamma coactivator 1-alpha (PGC-1α), resulting in adipose tissue hyperplasia following the MUFA diet or hypertrophic adipose after feeding on the SFA diet [41]. Ex vivo fatty acid treatments showed that a specific MUFA, palmitoleate, increases the adipose progenitor proliferation through increasing IGF-1 sensitivity [48]. Other pre-clinical studies show that while hypertrophy is strongly correlated with diet, hyperplasia adipose may be more dependent on the interaction between diet and genetics. The extent of hyperplastic expansion was dependent on genetic strain undergoing a high-fat diet [49]. Overall, adipose expansion mechanisms which cause hypertrophy or hyperplasia may be fatty acid-dependent, thus dictating the metabolic phenotype and potential tumorigenic microenvironment.

### 2.2. Obesity’s Role in Immune Cell Fractions and Function in Cancer

Adipose tissue was originally viewed solely for energy storage; however, it is now proven to have important endocrine functions including secreting cytokines and adipokines [9]. Adipose tissue is mainly composed of fat-storing adipocytes, which are enveloped by a stromal vascular fraction (SVF) composed of a diverse collection of cells including pre-adipocytes, fibroblasts, endothelial, and immune cells. The expansion of adipose tissue depots enhances immune cell infiltration and instigates more pro-inflammatory immune cell populations to further increase inflammation [10,11]. Adipose immune cell infiltration is probably instigated by hypertrophic adipocytes producing monocyte chemotactic protein-1 (MCP-1), which recruits pro-inflammatory cells [50,51]. With increasing adiposity, the SVF becomes enriched with macrophages, T cells, B cells, dendritic cells (DCs), invariant natural killer T cells (iNKT), mucosal-associated invariant T (MAIT) cells, gamma delta (γδ) T cells, and innate lymphoid cells [52]. The presence of these immune cells is integral to chronic low-grade inflammation development which is essential to metabolic disease and is believed to play an important role in obesity-related cancer risk [12,13].

With increasing adiposity, the presence of adipose tissue macrophages (ATMs) increases, paired with a phenotypic switch from anti-inflammatory M2-like (F4/80-) ATMs to pro-inflammatory M1-like ATMs (F4/80+) [50]. M1-macrophages are central cells in promoting inflammation in the adipose tissue microenvironment, specifically in obesity [53,54,55]. Furthermore, increased NEFA can enhance macrophage polarisation towards the M1-like phenotype [56]. Obese ATMs support tumorigenesis through IL-6 secretion, which promotes stem-like properties. Furthermore, weight reduction in a pre-clinical model reverses the macrophage reprogramming and oncogenesis [57]. ATMs from obese patients induce inflammation and lipid accumulation in cancer cells. Furthermore, tumour-associated macrophages have gene expression profiles more similar to obese ATMs versus lean ones [58].

T cell populations, specifically CD4+ and CD8+, are also changed in obesity. In the adipose, CD8+ T cells experience higher activation, while T cell subsets shift to a more pro-inflammatory phenotype with higher T helper (Th) subsets of Th1 and Th17 cells and lower levels of regulatory T (Treg) and Th2 cells [59]. Alternatively, tumour resident CD8+ T cells were reduced in tumours from obese mice. Additionally, tumour infiltrating CD8+ T cells were functionally and metabolically impaired with lower chemokine secretion and proliferation capability, resulting in a reduced ability to control tumour growth [60]. Metabolic plasticity has been identified as a pivotal regulator of T cell responses, with Treg cells exhibiting heightened employment of fatty acid oxidation (FAO), whereas effector cells preferably use glycolysis [61]. Remarkably, adipose tissue procured from viscerally obese patients have increased secreted levels of mediators related to Th17 immune responses. Th17 and Treg cell populations are balanced in the gastrointestinal system, resulting in normal immune system function and tissue homeostasis. At the gastric tumour site, Th17 and Treg cells infiltrate, proving that the tumour microenvironment could cause the Th17 and Treg cells to become imbalanced [62].

iNKT cells are a particular subgroup of T cells which are swiftly activated in response to excess lipids bound through CD1d, an antigen-presenting molecule which is expressed by DCs or macrophages [63]. Interestingly, CD1d cells are highly expressed in the omentum, an integral part of the VAT. However, the frequencies of these cells are depleted in the omental VAT of morbidly obese patients and cancer patients [64]. Due to the iNKT cell’s close interplay with lipid antigens, it is foreseeable that lipid profile modifications in the lipid profile of the tumour microenvironment would alter their immuno-modulatory effects. Increased lactic acid levels (which are indicative of glycolytic metabolism) in the tumour microenvironment have been implicated in reducing PPARγ on intratumoural iNKT, diminishing cholesterol synthesis and IFN-γ production and reducing their anti-tumour immunity efficacy [54]. However, the introduction of a PPARγ agonist combatted these effects and restored interferon gamma (IFN-γ) production [65]. This indicates the significance of the tumour microenvironment’s lipid profile to promote an effective anti-tumour immune response.

Following high-fat diet initiation, B cells infiltrate into the adipose tissue [66]. B cells secrete pro-inflammatory IL-6, IL-8, and TNFα while inducing other cells to secrete leptin and MCP-1, which are related to intracellular pathways that promote CRC growth and metastatic spreading [67,68]. Additionally, B cells can modulate T cell behaviour, perpetuating inflammation and insulin resistance [69,70,71]. Within the tumour microenvironment, B cells recruit and activate T cells which influence other immune cells to resist tumour cells [72]. Regulatory B cells (Bregs) produce anti-inflammatory IL-10, IL-35, and transforming growth factor—beta (TGF-β), which inhibits immunity resulting in promoting tumour growth [73]. Additionally, Bregs can deplete CD8+ T cells, further increasing the immunosuppression [74].

DCs play a fundamental role in antigen presentation and commencing the anti-tumour immune response, and have been identified as a prominent player in obesity-associated immune responses. DCs represent a significant proportion of infiltration cells during adipose expansion [75]. Additionally, high NEFA levels lead to lipid-loaded DCs with diminished antigen-presenting capabilities and a decreased capacity to effectively stimulate T cells [76]. Normal DC function is required for T cell-mediated tumour clearance. DC-dependent immunotherapy reduced the tumour size in lean mice but was greatly reduced in obese mice [77].

Myeloid-derived suppressor cells (MDSC) increase with obesity in mouse models and humans, in circulation and within adipose tissue [78,79]. Intriguingly, lipid accumulation at the tumour site has been related to metabolic plasticity in MDSCs, guiding them from a glycolytic phenotype towards the enhanced utilisation of FAO and oxidative phosphorylation. This metabolic preference shift confers MDSCs with enhanced immunosuppressive properties, leading to a diminished effect on anti-tumour immunity [80]. High-fat diet-enhanced MDSC accumulation results in increased tumour progression and metastasis through reduced T cell activation [81].

Neutrophils have been reported to be increased in morbidly obese humans [82]. Interestingly, within a glucose-limited tumour microenvironment, neutrophils use FAO to fuel ROS production and suppress T cells [83]. This metabolic plasticity has been implicated in aiding cancer growth, metastasis, and recurrence [84]. Additionally, a high-fat diet elevates levels of granulocyte-macrophage colony-stimulating factor (GM-CSF), which increases neutrophil presence and promotes tumour growth and metastasis [85].

Obese humans have diminished natural killer (NK) cell frequencies with decreased cytotoxicity, which may lead to impaired tumour immune responses [86]. A fatty acid-enriched microenvironment impairs NK cell functionality [87], validating the theory that obesity may have disadvantageous effects on NK cell performance. NK cells from obese cancer patients are recruited to adipose tissue where they undergo irreversible dysregulation leading to cell death. In OAC patients, higher NK cell frequencies have been reported within the VAT whilst diminished expression was detected within tumour tissue [88,89].

### 2.3. Diet and Fatty Acid’s Role in Immune Cell Fractions

Obesity-associated inflammation and metabolic perturbations are partly caused by the alterations in adipose immune cell phenotypes alluded to above and previously reviewed [52,90]. Recent evidence suggests that the extent of these changes can be dependent on the composition of fatty acids that cell populations are exposed to (Figure 2). Fatty acids can be sourced from the diet, as well as resulting from endogenous de novo lipogenesis in response to energy excess and fatty acid metabolism. The pro-inflammatory effects of SFA are well-characterised. Briefly, SFA can signal through a cytosolic lipid-responsive pattern recognition receptors protein complex, the NOD-, LRR-, and pyrin domain-containing protein 3 (NLRP3) inflammasome, promoting pro-inflammatory cytokine expression including IL-1β and IL-18. This occurs through a two-phase process requiring stress signals which can include cholesterol, NEFA, ATP, pathogens (e.g., lipopolysaccharide (LPS)), glucose, and ROS, all of which are increased in obesity [6]. The SFA palmitate activated the NLRP3 inflammasome in both macrophages and DCs in mice following a high-fat diet [41,91,92,93,94]. Additionally, a palmitate treatment increased TNFα and IL-1β secretion, paired with a decrease in anti-inflammatory cytokine IL-10 secretion [93,95]. Conversely, MUFA does not activate the NLPR3 inflammasome like SFA. MUFA reverses pro-inflammatory cytokine expression following a SFA diet. This can be attributed to a higher level of anti-inflammatory gene expression (IL-10, macrophage galactose N-acetyl-galactosamine specific lectin 2 (Mgl2), mannose receptor C-type 1 (Mrc1), Tgfb1) and a shifted macrophage phenotype towards M2, as displayed by an increase in the oxygen consumption rate [96].

In macrophages, palmitate has been reported to elevate pro-inflammatory response signals [97,98]. Palmitate increases the expression of NLRP3, nitric oxide (NO), IL-1β, IL-6, TNFα, nuclear factor kappa B (NF-κB), c-Jun N-terminal kinases (JNK), mitogen-activated protein kinase kinase (MKK)4/7, IL-10, MCP-1, IFN-γ, M1 polarisation, and CD36 [98,99,100]. In contrast, oleate has anti-inflammatory effects by inhibiting the pro-inflammatory responses driven by SFA steric acid [101], along with promoting M2-like polarisation in macrophages [102]. In T cells, it has been demonstrated that palmitate increases the expression of insulin receptors, ROS, and cytokines (IFN-γ, IL-1β, IL-2, IL-6, IL-8, IL-10, TNFα), and insulin receptor substrate 1 (IRS-1) generation and proliferation [103]. Additionally, palmitate increases the expression of PI3K/Akt, JNK, and extracellular signal-regulated kinase (ERK)1/2 pathways [104]. Alternatively, oleate decreases proliferation and pro-inflammatory cytokines (IL-2, IFN-γ) [105]. In B cells, palmitate reprogrammed B cells to be immunosenescent [106]. Oleate was required for normal mTOR activity and mitochondrial function, and to prevent endoplasmic reticulum (ER) stress [107]. Palmitate stimulates the pro-inflammatory secretion of IL-1β through toll-like receptor (TLR) activation in DCs [92,108]. In neutrophils, palmitate increases ERK1/2, Akt, ROS, and chemotaxis [109,110]. Oleate decreases migration while also increasing ERK1/2, Akt, NF-κB, ROS, IL-1β, IL-8, and ATP [111,112,113,114,115]. The replacement of palmitate with oleate can reduce pro-inflammatory cytokine secretion. Since palmitate and oleate have different effects on adipose location, expansion, and immune cell characteristics, they could also have differential effects on obesity-related cancer initiation and progression.

## 3. Metabolic Flexibility in Cancer Cells in the Tumour Microenvironment

Extensive metabolic reprogramming occurs in cancer to keep up with the increased energy demand and to obtain membrane materials for proliferation. Metabolic reprogramming of fatty acids is activated in cancer cells to support their increased bioenergetic demand [116]. These changes in fatty acid metabolism are often paired with mitochondrial dysfunction, which is common in cancer cells to aid tumour progression [117]. Aberrant cellular metabolism is an essential survival advantage induced by cancer to escape the cytotoxic effects of chemotherapy and chemoradiotherapy [118,119]. Lipid metabolism, particularly FAO, enhances treatment resistance in cancer cells through the upregulation of lipogenic or lipolytic enzyme expression [120]. Resistant cells often increase sterol regulatory element binding protein (SREBP)-induced de novo lipogenesis through fatty acid synthase (FASN), and the elongation of very long-chain fatty acid 6 (ELOVL6) or stearoyl-CoA desaturase 1 (SCD1) overexpression in tyrosine kinase inhibitor-resistant cells [121,122]. Additionally, mitogen-activated protein kinase pathway inhibitors increase FAO, which can result in drug resistance [123]. The exploitation of lipid metabolism and FAO is strongly elicited by metastatic cancer cells, M2 macrophages, memory CD8+ T cells, and tissue-resident Treg cells, specifically in VAT [124,125,126,127].

Another metabolic mechanism that cancer cells utilise is glucose metabolism reprogramming, as an innate adjustment in cancer cells. Glucose normally undergoes aerobic respiration, resulting in pyruvate production, which is further converted into acetyl-CoA. The conversion of acetyl-CoA to malonyl-CoA then enables the endogenous production of the SFA palmitate within the cell. From here, palmitate can be desaturated by SCD1 and converted into palmitoleate, or can be elongated by ELOVL6 into stearic acid and then desaturated by SCD1 to form the MUFA oleate. These fatty acids are stored in lipid droplets to be utilised for energy demands during times of cellular stress [128]. Remarkably, FASN is diminished in obesity [129,130], whilst FAO is elevated [131], indicating that obesity may encourage a metabolic shift resulting in the enhanced utilisation of lipid metabolism and FAO. Additionally, FASN is thought to modulate thresholds that trigger receptor signalling and ultimately regulate the balance between anti-proliferation and tumorigenesis [132]. FAO can also be activated by other upstream activators such as AMP kinase (AMPK), promyelocytic leukaemia-peroxisome proliferator-activated receptor (PML-PPAR) pathway, and glycolysis [133]. These cellular metabolic aberrations which increase cancer cell formation can be extended to lead to a metastatic phenotype.

Primary cancer cells rely heavily on glycolytic metabolism to grow and survive, prompting this metabolic dependence despite the oxidative stress prompted by radiation-induced inflammation to facilitate DNA damage repair [134]. However, metastatic cancer cells depend more on oxidative phosphorylation-associated metabolism and FAO [135,136]. Energy metabolism flexibility is critical in aiding cancer cells’ ability to undergo epithelial–mesenchymal transition (EMT) and migration to facilitate distant metastasis [137].

## 4. Obesity and Cancer Metastasis

Research is centred on the impact of obesity and its contribution to the metabolic changes and EMT that support cancer cells’ migratory capacity to develop distant metastasis. Metastasis encompasses a succession of transfiguring alterations in cancer cells including their metabolic preferences and plasticity, and in their surrounding stroma, which can be triggered as a response to cancer therapies. The phases entailed in the colonisation of distant metastasis include local invasion, intravasation, circulation of cancer cells, extravasation, and, lastly, the establishment of local and distant metastasis [138]. Obesity-linked cancers, including gastric and colon cancer, often metastasise to the omentum, which advocates the contribution of adipose tissue to the metastatic cascade [139]. Adipose tissue can drive this cascade through multiple mechanisms which include increased adipokine/cytokine secretion, metabolic reprogramming, and angiogenesis. An increased secretion of IL-6, leptin, IGF-1, and TNFα in obesity promotes EMT and inflammation, and dampens the immune response [2]. Furthermore, angiogenesis is promoted in obese adipose through an increase in the release of pro-angiogenetic TNFα, IL-6, and vascular endothelial growth factor (VEGF) [140]. These advancements position obese adipose tissue as a highly favourable site to facilitate the development of pre-metastatic niches [141]. Ultimately, adipose tissue can increase tumorigenesis and aid the metabolic flexibility required by cancer cells and metastasis.

## 5. Evidence Linking Obesity to Gastrointestinal Cancer Risk

**Figure 4:**
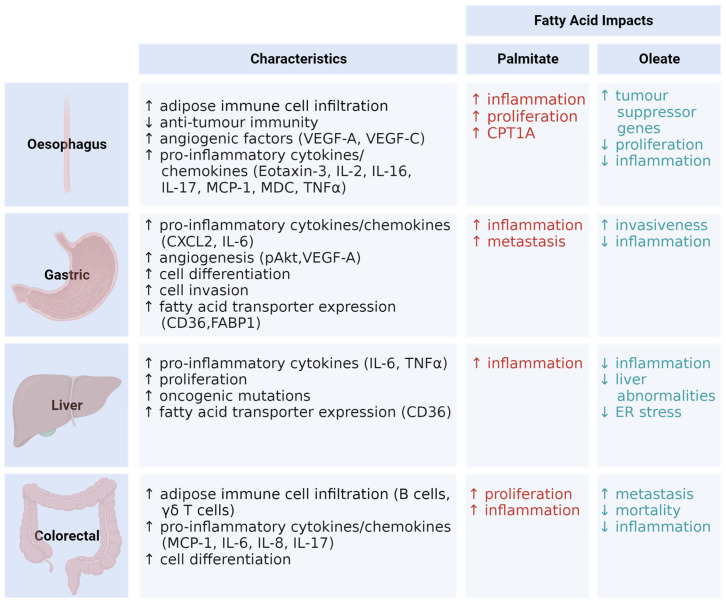
Obese adipose and fatty acid effects on gastrointestinal cancers. Obese adipose increases immune cell infiltration, which then creates a tumorigenic environment on neighbouring gastrointestinal organs including the oesophagus, stomach, liver, and colon. Effects seen are an increase in inflammation, angiogenesis, proliferation, and cell differentiation, which can differ based on the specific organ. Palmitate and oleate have differential effects on oesophageal, gastric, liver, and colorectal cancer. Generally, saturated fatty acid palmitate drives cellular behaviours which may increase a tumorigenic environment to a greater extent than monounsaturated fatty acid oleate. CPT1A, carnitine palmitoyltransferase 1A; CXCL, chemokine (C-X-C motif) ligand; FABP1, fatty-acid binding protein 1; IL, interleukin; MCP-1, monocyte chemoattractant protein-1, MDC, macrophage-derived chemokine; TNFα, tumour necrosis factor alpha; VEGF, vascular endothelial growth factor; ↑, increase; ↓, decrease. This figure was created using Biorender.com (accessed on 7 August 2023).

Adipose tissue expansion, specifically VAT, can affect gastrointestinal tract cancers due to anatomical proximity. Upper gastrointestinal cancers such as oesophageal tumours are exposed to acid reflux and bile acid following excess dietary fat ingestion [142]. Lower gastrointestinal cancers, including CRC, are surrounded by VAT, increasing the exposure to a sub-acute pro-inflammatory environment [143]. The dysregulation of immune cells in the adipose tissue, discussed above, may also create an environment that drives tumorigenesis. Increasing adipose inflammation with obesity disrupts tissue homeostasis, hampers immunological responses, and can lead to tissue hyperplasia or death, ultimately creating obesity-driven tumorigenesis [143,144,145,146]. Cytokines that are classically upregulated in obesity, such as TNFα, IL-6, and TGFβ, promote tumour cell proliferation and invasion, and possible tumour formation [147,148]. Furthermore, the phenotypic changes described above in CD4+ and CD8+ cells are involved with tumour growth and metastasis proximal to adipose tissue [149]. Obese adipose tissue immune cell dysfunction creates an environment which allows tumorigenic cell growth and metastasis. The culmination of this results in obesity instigating cancer including OAC, gastric, liver, and CRC (Figure 4).

### 5.1. Obesity and OAC

Large-scale epidemiological studies consistently illustrate a compelling association between the risk of cancer onset or progression and increased BMI for numerous gastrointestinal cancers including OAC. OAC is among one of the cancer types most strongly correlated with escalating obesity levels [150,151,152,153,154], making it an exemplary model for studying obesity’s influence on cancer, especially because of its proximity to VAT depots. Interestingly, oesophageal cancer adipose tissue has been reported to recruit immune cells while negatively impacting their function, thereby enhancing anti-tumour immunity [88,155,156,157]. Previous research has reported that various pro-inflammatory mediators in the circulation and expressed within the tissue have shown associations with clinical outcomes in OAC, particularly factors that are involved in the recruitment and activation of innate immune cells [158]. Adipose tissue energy metabolism and the impact of its secretome on cancer cell metabolism is an emerging area of research. In OAC patients, VAT had higher oxidative phosphorylation compared to SAT. Additionally, VAT secretions increase angiogenic and inflammatory cytokines including VEGF-A, VEGF-C, IL-2, IL-16, and TNFα [159]. Viscerally obese OAC patients with increased oxidative phosphorylation were correlated with metabolic dysfunction and increased pro-inflammatory mediators IL-5 and IL-7. Furthermore, glutamine levels are reduced while its metabolised product glutamate’s levels are increased in the adipose secretome of obese compared to non-obese OAC patients [160]. Recent research has indicated that the secretome of adipose explants derived from OAC patients is altered due to increased visceral adiposity [157]. Interestingly, inflammatory factors including Eotaxin-3, MCP-1, macrophage-derived chemokine (MDC), and IL-17 were shown to be increased in the adipose secretome of patients with enlarged VAT depots. Previously, these factors have been shown to increase immune cell infiltration [161] and may be linked with maintaining the low-grade inflammatory state that is associated with obesity.

### 5.2. Obesity and Gastric Cancer

Overweight and obesity cause approximately 6% of gastric cancer (GC) [162]. Furthermore, GC metastasis is commonly directed towards the VAT depot, highlighting the important role adipose plays in tumour progression. The human gastric adenocarcinoma cell proliferation and migration rate increased following incubation with human visceral adipose-conditioned media (ACM). Additionally, the S phase population of the cell cycle was increased. Furthermore, GC cells cultured with human visceral ACM were injected into nude mice, which increased the rate of tumour growth compared to cells not grown in ACM [163]. In vitro, the co-culture of adipocytes with GC cells drove the adipocytes to dedifferentiate into cancer-associated fibroblasts, with increased IL-6 secretion. Furthermore, VAT proximal to primary tumours displayed reduced adiponectin levels in patients who exhibited subserosal or serosal invasion [164]. Visceral ACM induced the angiogenesis of GC cells through Akt phosphorylation and overexpression of VEGF-A with increased secretion of chemokine (C-X-C motif) ligand (CXCL) 2 [165]. The increased expression of fatty acid transporters such as CD36 and fatty acid-binding protein 1 (FABP1) are increased in obesity [166,167] and drive increased pathway expression, which increases GC metastasis. This highlights the effects of obese adipose on the tumorigenic environment in the stomach.

### 5.3. Obesity and Liver Cancer

Liver cancer, or hepatocellular carcinoma (HCC), has a constantly increasing trend in the USA and many European countries. HCC can arise from liver cirrhosis, credited to hepatitis B and C virus infections and/or heavy alcohol intake [168]. The increase in HCC is paralleled with an increase in NAFLD. One study found that NAFLD, in the absence of obesity, elevated the cancer risk primarily in the liver, gastrointestinal tract, and uterus [169]. Conversely, approximately 23% of HCC cases in the UK are caused by overweight and obesity [162]. In male HCC patients, the VAT depot mass was higher in HCC versus non-HCC patients and it was a risk factor for the recurrence of HCC after liver transplantation [170]. Adipose exclusively secretes adiponectin, an adipokine which reduces triglycerides levels and controls insulin signalling, whose levels are decreased in obesity [171]. Interestingly, adiponectin secretion is positively correlated with a poor prognosis of liver cancer [172]. This may indicate that obesity alone is not the only determinant factor in HCC prognosis. Inflammatory adipose-derived cytokines, including TNFα and IL-6, are oncogenic signalling molecules in liver cancer [173]. Pro-inflammatory activities induced by the adipokine leptin and lipotoxicity, reflecting increased fatty acid storage that spills over or escapes from adipose, increase the proliferation and oncogenic mutations resulting in carcinogenesis in the liver [174]. The second most prevalent liver tumour is cholangiocarcinoma, which occurs within the bile duct epithelium [175]. A high BMI is significantly associated with increased tumour size and metastasis rates leading to a poor prognosis and a heightened risk of recurrence. Furthermore, the tumour tissue from obese patients displayed altered immune characteristics which included increased PD-L1 expression, decreased CD8+ T cells, and increased FOX p3 + T cells [176]. Ultimately, the obese adipose phenotype has significant effects on liver health and can perpetuate hepatic cancer formation.

### 5.4. Obesity and CRC

CRC is a predominantly obesity-associated cancer which is strongly associated with lifestyle factors such as diet [177]. Obese-associated inflammation can promote CRC. Mutagenesis can occur through increased reactive oxidative damage and epigenetic silencing [142,178]. Increased IL-6 production can shift intestinal macrophages towards a M2-like macrophage phenotype in mouse models which overlaps with tumour-associated macrophages [179]. These M2-like macrophages then recruit B cells and γδ T cells to the tumour environment. CC motif chemokine receptor 6 (CCr6)-expression γδ T cells secrete IL-17, which further increased the colon inflammation in a T cell receptor alpha (TCRα) −/− mouse model [180]. When the recruitment of B cells and γδ T cells was blocked, there was a suppression of CRC development [181]. ACM from obese patients and lean and obese CRC subjects released more IL-6, IL-8, and MCP-1 compared to healthy lean subjects. This ACM was then cultured on DCs, promoting the differentiation and increased expression of programmed death-ligand 1 and 2 (PD-L1, PD-L2) with a diminished IL-12/IL-10 ratio, thus preventing DC-mediated γδ T cell activation [182]. While obesity may drive CRC development, it can also have differing effects on disease outcomes. There is a negative impact of BMI concerning disease relapse and death in stage III patients with a BMI > 30 km m^2^ [183]. Alternatively, there was also the emergence of the obesity paradox. Immune checkpoint therapy has been reported to have a positive association with obesity [184]. This highlights the ambiguous nature of obesity-driven cancers and therapies, which requires a greater understanding of disease mechanisms leading towards a personalised response.

## 6. SFA and MUFA’s Roles in Gastrointestinal Tumorigenesis

Lipids are emerging as key molecules fuelling cancer cell proliferation. The nutritional modulation of dietary fat is now thought to be important in cancer; however, little is known about how individual dietary lipids may regulate tumour growth and metastasis. The overconsumption of dietary fat may be positively or negatively correlated with cancer risk, depending on the fatty acid and cancer type. In vitro, the SFA lauric acid suppressed CRC cell proliferation, while in vivo, a palmitate-rich high-fat diet stimulated tumour growth. In vitro, MUFA oleate promoted growth in colon cancer cell lines while suppressing the growth and survival of GC cells [185]. Research into the role of dietary SFA and MUFA in tumorigenesis is increasing. Cell proliferation pathways, including ERK1/2-mTOR-NF-κB and PI3K/Akt, were differentially modulated by SFA and MUFA in a model- and cancer-dependent manner [185], suggesting that different dietary lipids may have distinct effects in tumorigenesis.

There is some evidence, albeit limited, that fatty acids may have distinct effects on gastrointestinal cancers. Palmitate upregulated carnitine palmitoyltransferase 1A (CPT1A) in the disease sequence from Barrett’s oesophagus to OAC, in both in vitro and mouse models, resulting in increased cell proliferation [186]. Importantly, CPT1A is a rate-limiting enzyme in FAO, whose substrate is palmitate, which has been linked with promoting cancer cell proliferation [187]. Oleate downregulated cell proliferation in OE19 and OE33 oesophageal cancer cell lines through the increased phosphorylation of AMPK with reduced S6 activation. Additionally, oleate increased the expression of tumour suppression genes p53, p21, and p27 [188]. Palmitate promoted metastasis both in vitro and in vivo through CD36 receptor activity and via the AKT/glycogen synthase kinase 3 beta (GSK3β)/β-catenin pathway [24]. A palmitate treatment also promoted gastric metastasis through the fatty-acid binding protein 5-specific protein 1-urothelial cancer-associated 1 (FABP5/SP1/UCA1) pathway [189]. In vitro, a co-culture of GC cells with isolated omental adipocytes showed an increase in oleate within the gastric cell. An oleate treatment on gastric cells enhanced the invasiveness through the PI3K/Akt pathway [190]. In human hepatoma cells, palmitate disturbed lipid metabolism and increased the protein expression of NLRP3 inflammasome and ER stress, while oleate was able to rescue these cells from pyroptosis. In vivo regression studies showed that the replacement of a high-fat diet with an oleate-rich olive oil reduced liver abnormalities and inhibited ER stress [191]. In vitro, the administration of palmitate to CRC cells increased proliferation through an enhanced expression of β2-adrenergic receptors, which are vital for CRC growth [192]. An in vitro palmitate treatment of intestinal organoids increased the number of leucine-rich repeating-containing receptor 5 (Lgr5^+^) intestinal stem cells in a PPAR-d dependent manner, which boosted their ability to form colorectal adenocarcinomas [193]. High-fat diet feeding in CRC mice showed an increase in palmitate, which increased the beta-2 adrenergic receptor (β2AR) expression and β-adrenergic signalling pathway, which was then reduced with the removal of the high-fat diet [192]. Furthermore, a short-term palmitate-rich diet induced a more aggressive tumour cell profile that endured as cellular memory in a CD36-dependent manner [194]. The blockage of CD36 expression inhibited metastasis, highlighting that dietary fatty acids are needed to promote metastasis (Figure 4). Whilst most of the data discussed above pertain to in vitro fatty acid exposures and pre-clinical in vivo diet-induced mechanisms, we need greater translational data to understand if, ultimately, diets rich in saturated fat, such as palmitate, may contribute to cancer development to a greater extent than unsaturated fat, including oleate.

## 7. Conclusions

There are clear associations between obesity and gastrointestinal cancers. However, obesity is extremely heterogeneous and highly dictated by adipose structure, immune cell fractions, and dietary components. Hypertrophic adipose mass recruits more pro-inflammatory immune cells compared to hyperplastic adipose mass. Furthermore, visceral adipose has more pro-inflammatory behaviour and fatty acids released compared to subcutaneous adipose, which exacerbates cellular metabolic dysfunction. The importance of dietary fats, specifically SFA and MUFA, on the adipose function and extent of inflammation is potentially evident. SFA increases hypertrophic inflammatory adipose microenvironments, creating ideal pre-metastatic niches for tumorigenesis to occur. Alternatively, MUFA does not display these same effects. Furthermore, the different effects palmitate and oleate have on tumorigenesis in gastrointestinal cancers highlight the importance of the specific type of fatty acid intake on cancer initiation and/or progression. However, further research on the difference between SFA and MUFA in gastrointestinal cancers is required to fully elucidate the mechanisms that differ between these fatty acids. This will give us greater translation insight with respect to the true impact of dietary fat intake and risk. With this enhanced knowledge base, hopefully we can develop dietary preventions and/or interventions. These will rely on a more targeted understanding of obesity and the nutrition environment, embracing a precision nutrition approach which may be a more effective line of treatment. Both obesity and cancer are highly diverse and individualised diseases on their own, and their complexity may be amplified when they are combined. Precision nutrition therapy could target individual conditions to maximise the effectiveness and is a promising tool in cancer therapy.

## Glossary

ACM	adipose-conditioned media
AMPK	AMP-activated protein kinase
ATM	adipose tissue macrophage
ATP	adenosine triphosphate
β2AR	beta-2 adrenergic receptor
BMI	body mass index
Breg	regulatory B cells
CCr6	CC motif chemokine receptor 6
CPT1A	carnitine palmitoyltransferase 1A
CRC	colorectal cancer
CVD	cardiovascular disease
CXCL	chemokine (C-X-C motif) ligand
DC	dendritic cell
ELOVL	elongation of very-long-chain fatty acid
EMT	epithelial–mesenchymal transition
ER	endoplasmic reticulum
ERK	extracellular signal-regulated kinase
FABP1	fatty-acid binding protein 1
FABP5/SP1/UCA1	fatty-acid binding protein 5-specific protein 1-urothelial cancer associated 1
FAO	fatty acid oxidation
FASN	fatty acid synthase
γδ	gamma-delta
GC	gastric cancer
GLUT4	glucose transporter type 4
GM-CSF	granulocyte-macrophage colony-stimulating factor
GSK3β	glycogen synthase kinase 3 beta
HCC	hepatocellular carcinoma
IFN-γ	interferon gamma
IGF-1	insulin-like growth factor 1
IL	interleukin
iNKT	invariant natural killer T cells
IR	insulin resistance
IRS-1	insulin receptor substrate 1
JNK	c-Jun N-terminal kinases
Lgr5+	leucine-rich repeating-containing receptor 5
LPS	lipopolysaccharide
MAIT	mucosal-associated invariant T cells
MAPK	mitogen-activated protein kinase
MCP-1	monocyte chemoattractant protein-1
MDC	macrophage-derived chemokine
MDSC	myeloid-derived suppressor cells
Mgl2	macrophage galactose N-acetyl-galactosamine specific lectin 2
MKK	mitogen-activated protein kinase kinase
Mrc1	mannose receptor C-type 1
mTOR	mechanistic target of rapamycin
MUFA	monounsaturated fatty acid
NAFLD	non-alcoholic fatty liver disease
NEFA	non-esterified fatty acid (or free fatty acid)
NF-κB	nuclear factor kappa B
NK	natural killer
NLRP3	NOD-, LRR- and pyrin domain-containing protein 3
NO	nitric oxide
OAC	oesophageal adenocarcinoma
PD-L	programmed death-ligand
PGC-1α	peroxisome proliferator-activated receptor gamma coactivator 1-alpha
PI3K	phosphoinositide-3-kinase
PML-PPAR	promyelocytic leukaemia-peroxisome proliferator-activated receptor
PPARγ	peroxisome proliferator-activated receptor gamma
RAS	rat sarcoma
ROS	reactive oxygen species
SAT	subcutaneous adipose tissue
scd1	stearoyl-CoA desaturase
SFA	saturated fatty acid
SREBP	sterol regulatory element binding proteins
SVF	stromal vascular fraction
T2D	type 2 diabetes
TAG	triacylglycerol
TCRα	T cell receptor alpha
TGF-β	transforming growth factor beta
Th	helper T cells
TLR	toll-like receptor
TNFα	tumour necrosis factor alpha
Treg	regulatory T cells
VAT	visceral adipose tissue
VEGF	vascular endothelial growth factor
